# Breast milk donation after neonatal death in Australia: a report

**DOI:** 10.1186/s13006-014-0023-4

**Published:** 2014-11-29

**Authors:** Katherine E Carroll, Brydan S Lenne, Kerri McEgan, Gillian Opie, Lisa H Amir, Sandra Bredemeyer, Ben Hartmann, Rachel Jones, Pieter Koorts, Helen McConachy, Patricia Mumford, Jan Polverino

**Affiliations:** Faculty of Arts and Social Sciences, University of Technology Sydney, PO Box 123, Sydney, NSW 2007 Australia; Faculty of Health Sciences, Mayo Clinic, Harwick 2, 200 First St SW, Rochester, MN 50091 USA; Mercy Hospital for Women, 163 Studley Rd, Heidelberg, Melbourne, VIC 3084 Australia; Judith Lumley Centre, La Trobe University, 215 Franklin St, Melbourne, VIC 3000 Australia; Royal Women’s Hospital, Parkville, Melbourne, VIC 3053 Australia; Royal Prince Alfred Hospital, Missenden Rd, Camperdown, NSW 2050 Australia; King Edward Memorial Hospital, 374 Bagot Rd, Subiaco, WA 6008 Australia; School of Pediatrics and Child Health, The University of Western Australia, 35 Stirling Hwy, Crawley, WA 6009 Australia; Royal Brisbane and Women’s Hospital, Butterfield St, Herston, Brisbane, QLD 4006 Australia

**Keywords:** Breast milk donation, Infant death, Bereavement, Human milk bank, Neonatal intensive care

## Abstract

Lactation and breast milk can hold great value and meaning for grieving mothers who have experienced a recent death of an infant. Donation to a human milk bank (HMB) as an alternative to discarding breast milk is one means of respecting the value of breast milk. There is little research, national policy discussion, or organizational representation in Australia on the subject of breast milk donation after infant death. On 29 November 2013 the Mercy Hospital for Women in Melbourne, Australia hosted Australia’s first National Stakeholder Meeting (NSM) on the topic of milk donation after neonatal death. The NSM drew together representatives from Australian HMBs, neonatal intensive care units (NICUs) currently using donor human milk, and Australia’s chief NICU parent support organization. The NSM was video-recorded and transcribed, and analyzed thematically by researchers. This article reports the seven dominant themes discussed by stakeholders during the NSM: the spectrum of women’s lactation and donation experiences after infant death; the roles of the HMB and NICU in meeting the needs of the bereaved donor; how bereaved mothers’ lactation autonomy may interface with a HMB’s donation guidelines; how milk donation may be discussed with bereaved mothers; the variation between four categories of milk donation after neonatal death; the impact of limited resources and few HMBs on providing donation programs for bereaved mothers in Australia. This article provides evidence from researchers and practitioners that can assist HMB staff in refining their bank’s policy on milk donation after infant death, and provides national policy makers with key considerations to support lactation, human milk banking, and bereavement services nation-wide.

## Background

The neonatal intensive care unit (NICU) provides a unique context for research on human milk donation. In Australia, where this research was conducted, it is common for most NICU mothers to initiate milk expression, express during the hospital stay, store expressed breast milk (EBM), and to provide breast milk and/or breastfeed on discharge from NICU [1]. As a consequence, some NICU mothers will also donate EBM to a human milk bank [2], either during their infant’s NICU admission, or after discharge. Australia currently has 22 NICUs, of which six receive pasteurised donated breast milk from one of Australia’s five HMBs [3].

Tragically for many families, despite the medical interventions provided, not all NICU infants survive. In this article we report on the main issues discussed during Australia’s first National Stakeholder meeting (NSM) on the topic of breast milk donation after neonatal death, which drew together representatives from Australian HMBs, NICUs currently using donor human milk, and Australia’s chief NICU parent support organisation, ‘Miracle Babies Foundation’. In 2011, 7,412 babies (2.5% of notified live births) were admitted to one of Australia’s 22 level III NICUs, with a mortality rate of just over 5% [1]. In cases of infant death, it is of utmost importance that a mother’s lactation management and breast care are attended to by skilled health professionals in a timely manner [4-6], including what to do with existing stores of breast milk that the bereaved mother may have in the NICU freezers and in her home. This issue is pertinent in light of human milk banking as bereaved mothers may be willing to donate breast milk after infant death, either as a frozen store or as a result of expressing milk during lactation suppression [2,4,7].

Research, national policy discussions, and organisational representation in Australia on the subject of breast milk donation after infant death and the role of HMBs are scarce. In 2007 Australia’s House of Representatives Standing Committee on Health and Ageing published a report on their inquiry into the health benefits of breastfeeding. It highlighted three important issues associated with donation after bereavement: the despair felt by mothers who have excess milk and dispose of it because they are unaware of donation options; the wastage of milk that could otherwise be donated; and the positive feelings associated with donation [8]. The report emphasises the need to create opportunities for mothers to donate their milk as a precious resource. Seven years later, in 2014, The Commonwealth Department of Health released an issues and background paper on Donor Human Milk banking in Australia [3]. Although the incidence of neonatal death was covered, and a range of ethical issues associated with donation was discussed, the cohort of bereaved mothers as potential *and* actual donors of milk was overlooked. A review of Australia’s HMB websites similarly reveals that donors are predominantly characterised as women who have stored milk they wish to donate, women who are breastfeeding and wish to donate excess breast milk, or women who are pregnant and plan to donate excess once they have given birth. Australian HMBs are yet to develop coordinated protocols on milk donation after neonatal death that may assist in developing national resources and discussions across the various NICUs, HMBs and maternity services. However, at the local level, Australian milk banks are beginning to develop their own guidelines or “best practice” with regards to bereaved donation. This stands in contrast to discussion in the United States of America (USA) about the donation of breast milk to a HMB after neonatal death in both research [4,9] and organizational forums [2,7,9]. Many individual HMBs in the USA provide information about donation programs for bereaved mothers [10-12], and The United Kingdom Association of Human Milk Banks (UKAMB) features a bereaved donation program on their donation homepage [13].

Despite the awkwardness, silence and grief that surrounds infant death [14], lactation and breast milk hold great value and meaning for many bereaved mothers [4]. Donation to a HMB is one means of respecting the value assigned to lactation [6] and to breast milk [4]. This value was recently made evident in Australia’s social media. One bereaved mother posted a photo of the bottles of milk she donated on the Miracle Babies’ Facebook page which, as at 17 July 2014, had attracted 4893 ‘likes’ , and 193 ‘shares’ , and 533 ‘comments’ [15]. We use this example to demonstrate that bereaved donation is becoming more visible within the wider community: women are willing to share their personal experiences through Facebook, blogs [7] or online newsletters [2], and these experiences, in turn, are ‘shared’ and commented upon by others, thus broadening their reach and audience. It also suggests community support for more formal policy conversations on milk donation after bereavement among HMBs on a national level.

Lactation and donation choices are highly personal and variable. Some women choose to immediately suppress their lactation, while others continue to lactate for a period of time, during which some may also express their milk with the intention of donating [6]. Providing women with a choice of what to do with their lactation can be empowering at a time of grief [6]. For some women, lactation and milk donation after infant death acknowledges their motherhood status that may otherwise be denied [2,14]. For other women, milk donation may be a means through which they can memorialise their infant’s life by donating bodily substances that are directly connected with the life of their infant [14]. We now report upon the current practices and practice issues experienced by leading Stakeholders in Australia who work with donor human milk on a daily basis or with lactating women and their medical care.

## The national stakeholder meeting

On 29 November 2013 the Mercy Hospital for Women (Melbourne, Australia) hosted Australia’s first National Stakeholder Meeting (NSM) on the topic of milk donation after neonatal death. The NSM was scheduled to cover several topics related to milk donation after neonatal death (Table 1). The aim of the NSM was (i) to review and discuss research into milk donation after neonatal death in light of current practices in Australian HMBs and NICUs, and (ii) to write a publically accessible, peer-reviewed document that could inform and promote discussion about milk donation after neonatal death. Milk donation after infant death is a complex topic. This article discusses the content of the NSM and describes the five key considerations voiced by Stakeholders.

**Table 1:** **Structure of the National Stakeholder Meeting**

**Segment of NSM**	**Breakdown of timing of each segment at NSM**	**Details/discussion**
*Introduction*	20 mins	Stakeholders stated their institutions’ current position with regard to neonatal death and milk donation and any important personal experiences on the topic.
*Literature review*	15 mins	A literature review on lactation and milk donation after neonatal death was presented
*Research findings*	20 mins	Preliminary findings from part one of the current research project (‘Breast milk Donation After Neonatal Loss’) exploring bereaved mothers’ experiences with lactation and milk donation were presented
*Sociological and bioethical review*	15 mins	Key sociological and bioethical principles regarding milk donation after neonatal death were presented.
*Stakeholder discussion*	120 mins	Following the presentations, an interactive discussion among the Stakeholders was facilitated by an experienced social science researcher (KEC) to respond to the question, ‘what are the key considerations in our current practice, and in response to the NSM presentations that we need to consider for Australian HMBs?”

The NSM forms part of a research project titled, ‘Breast milk Donation After Neonatal Loss’ funded by the University of Technology, Sydney Australia, and conducted at two Level III Australian NICUs (2013–2014). The first part of the research project involved semi-structured qualitative interviews with bereaved mothers to ascertain their experiences of lactation during a NICU admission and after neonatal death, in addition to their preferences regarding milk donation. The experiences of women in our qualitative study were analysed and preliminary findings (forthcoming) were presented at the NSM to ensure the cohort of bereaved mothers’ voices were heard. We recognise that fathers’ support of their partners’ lactation and donation is important and an area that is ripe for future study.

The structure of the five-hour NSM centred on the expertise of all Stakeholders as clinical practitioners, researchers, NICU family representatives, and advocates in the area of milk donation, milk banking or lactation. Leading Australian Stakeholders were identified by the research team, and invited to attend the NSM. In total, 16 Stakeholders were invited, with 14 accepting invitations to attend the meeting, and 12 Stakeholders actually in attendance on the day. These 12 Stakeholders came from the four States in Australia with HMBs: New South Wales (NSW), Victoria, Queensland and Western Australia. A representative from the fifth Australian HMB, the Mothers’ Milk Bank (Tweed Heads, NSW), was invited to participate in the NSM, but was unable to attend. Those in attendance included the following categories: employees of Australia’s HMBs (n =8), health professionals currently practising in Australia’s milk-bank affiliated NICUs (n =4), breastfeeding researchers and academics (n =3), and the Director and Founder of Australia’s largest NICU family-patient support organisation, “Miracle Babies” (n =1).

The NSM was video-recorded in its entirety for transcription purposes. Human Research Ethics Committee (HREC) approval was granted by University of Technology, Sydney HREC (Ref#: 2013000270) and Mercy Hospital for Women HREC (R13/12). Both verbalisations and non-verbal gestures (such as nodding head, shaking head) were transcribed by one of the researchers (BSL). The transcription was analysed thematically by researchers (KEC and BSL). Non-verbal gestures in addition to spoken word formed the basis for analysis points of agreement, or difference in opinion among Stakeholders. Where this document refers to “agreement” amongst Stakeholders, this is based upon both nodding (gestures) and/or verbalised agreement amongst the group at the NSM (transcription), in addition to the editing process that was undertaken by each Stakeholder in the production of this document. All Stakeholders were invited to contribute to this published document. Quotations with ellipsis show omission of some words for brevity and relevance.

## Discussion: the stakeholders’ opinions

The NSM opened with Stakeholders presenting their NICU or HMB policy or current practice regarding milk donation after neonatal death. This presentation of current policy by Stakeholders is summarised in Table 2.

**Table 2:** **The NSM participating human milk banks**

**Milk bank**	**Website**	**Location**	**Operational since**	**Policy on bereaved milk donation at the time of the Stakeholder meeting**
PREM bank	http://www.kemh.health.wa.gov.au/services/PREM_Bank/	Perth, Western Australia	2006	● Since establishment PREM Bank have accepted donations of breast milk from bereaved families.
				● The policy was developed over time with input from the hospital’s Perinatal Loss Service, the Medical Director of NICU, the director of the human milk bank, and parents who have experienced infant death and lactation.
				● The policy acknowledges the individual differences in a grieving experience, and that some women may not want to actively suppress lactation after the death of their infant.
				● Donations to the milk bank are supported as a mother progresses toward involution (the physiological process that occurs when milk removal from the breast ceases) in the days or weeks following the death of her infant.
				● The policy also supports donations of previously expressed milk.
				● The bereaved donor also meet all other screening requirements expected of breast milk donors.
				● Donors who do not wish to undertake the full screening process may give consent for their donation to be used in research projects.
Royal Prince Alfred (RPA) Hospital Human Donor Milk Program (HDM)		Sydney, New South Wales	2005	● Does not have a policy relating to breast milk donation after neonatal death.
				● RPA Hospital HDM Program can only accept breast milk donations from mothers with infants in the NICU.
				● The RPA Hospital HDM Program has accepted donations of stored frozen breast milk from bereaved mothers whose babies have passed away in the RPA NICU
				● RPA NICU has not accepted milk from bereaved mothers who have birthed elsewhere, but have received inquiries from these mothers.
				● RPA staff does not approach bereaved mothers for breast milk donation. Rather, the mothers themselves approached RPA staff and offered their milk supply for donation.
				● The bereaved donor also meets all other screening requirements expected of breast milk donors.
Mercy Health Breast Milk Bank (MHBMB)	http://www.mercyhealthbreastmilkbank.com.au	Melbourne, Victoria	2011	● MHBMB does not have a specific policy with respect to breast milk donation after neonatal death.
				● Approaches made to the MHBMB by bereaved mothers are individually considered.
				● If donors with living infants experience neonatal death they may continue to donate.
				● Due to current practice restrictions, MHBMB is unable to accept donations of expressed breast milk collected prior to donor screening.
				● MHBMB can only accept breast milk donations from mothers who birthed at Mercy Hospital.
				● The bereaved donor also meet all other screening requirements expected of breast milk donors.
Royal Brisbane and Women’s Hospital (RBWH) Milk Bank	http://www.rbwhfoundation.com.au/index.php?option=com_content&view=article&id=224&Itemid=242	Brisbane, Queensland	2013	● The RBWH milk bank does not have a formal policy regarding milk donation after neonatal death. However, the RBWH milk bank was prompted to open a few weeks ahead of schedule due to a large donation of milk from a bereaved mother.
				● The RBWH milk bank supports breast milk donation after neonatal death and actively offers the option of donation to bereaved mothers.
				● The bereaved donor also meet all other screening requirements expected of breast milk donors.

Five themes arose from the coding of the NSM transcripts. These are each discussed in turn and represent the key talking points amongst the Stakeholders at the NSM. Each of the five themes is introduced by exemplary quotes that emerged from the NSM discussion and act to illustrate the diversity of opinions present amongst the Stakeholders.

(i) **The spectrum of milk donation after neonatal death**

“*I’ve been thrown into (*sic*) the deep end …so I find you really just have to listen and follow the parents, and every single one is different*.”

“*I am also concerned about where do the [bereaved] mothers fit, if they don’t fit our [donation] criteria?*”

“*Every situation is different and complex, and involves something we don’t really expect*.”

The Stakeholders recognised the complexity of the issue of bereaved donation and acknowledged that “one size does not fit all”. Stakeholders find themselves in a unique set of circumstances: there is a lack of precedent, guidelines, research and testing regarding milk donation and bereaved mothers. The Stakeholders used words such as “trial and error”, “ad-hoc basis” “thrown in the deep end” to describe their experiences in this field. The Stakeholders recognised that clinicians and lactation support staff will be working with differences with each individual case, and that it is vital these differences are catered for and recognised. Stakeholders suggested those approaching the bereaved mother in the early stages of neonatal death be sensitive to these differences and avoid judgement about what is a “normal” response to bereavement.

(ii) ** Caring for the bereaved donor**

“*Your concern is for that mother, I mean, more than, ‘Oh wow, I can get some milk here.’ You’re concerned about where she’s at.*”

“*It’s not about long term milk donation. It’s not about the milk bank. It’s about a service that we can provide; to provide some positive in that very terrible situation. So that’s our only role in that*.”

“*So in all of this I am kind of worried that we’re thinking we’re helping out but we’re not. We miss their issues completely*.”

Stakeholders voiced a tension associated with providing care for bereaved mothers who may also be milk donors. As clinicians, Stakeholders expressed that their “first and foremost” concern was for the mother’s wellbeing. They identified *three* additional points of care potentially required by bereaved mothers as milk donors. First, and perhaps the most unanimous point of additional concern was for the psychological and physical wellbeing of the bereaved mother. Although those in NICU attend to this care, it is also a time where discussions may be had about lactation management, including how to handle the breast milk that is expressed after neonatal death. The Stakeholders identified that lactation management and suppression should be discussed with the mother within four hours of the infant’s death, or possibly even sooner if the withdrawal of treatment is expected. Stakeholders identified this specific time period because of the frequency with which the mother may have been expressing her milk up until the death of her infant. This initial discussion of lactation management was seen to be relevant to donation after neonatal death because the care provider could be called upon to provide advice about what to do with the breast milk, including the provision of information on the option of donating frozen stores or freshly expressed milk to a HMB. Stakeholders agreed that ignoring breast milk donation in instances where it is an available option to women is undesirable practice as it means full lactation options were not provided. This includes the potential for offering lactation suppression medication too quickly without a chance to consider alternative management.

Second, the Stakeholders questioned the issue of duty of care. They discussed issues such as, “who should be responsible for the wellbeing of the bereaved mother?” and “Who is responsible for providing her with support regarding lactation and her options?” In current practice the responsibility for lactation management and potential donation is largely assumed on an ad-hoc basis, and this raised concern that bereaved mothers may be left without support or basic information on lactation suppression and donation. Stakeholders agreed that who, when and how bereaved mothers are approached regarding lactation management and donation options needs to be made operationally clear by each NICU and milk bank.

Third, some Stakeholders agreed that while they may be able to take some responsibility for the bereaved mother’s psychological wellbeing (such as having a conversation with her while she drops off her donated milk and signs the consent form); they could not take on full responsibility. Thus Stakeholders raised the importance of the community resources available to bereaved mothers, such as General Practitioners (GPs) to whom most postnatal women are advised to visit for a 6-week check. The involvement of GPs, however, leads to further considerations such as additional training and resources provided for GPs about the issue of lactation management, and bereavement. Other agencies that may provide bereavement care, but not necessarily relating to lactation were identified by Stakeholders and include community nurses, child health nurses, social workers, obstetricians, and perinatal loss services such as “SIDS and Kids”

(iii) ** Women’s autonomy**

“*I know that this is a particular group [bereaved mothers], but there are a lot of the parallels with this group of women and all of our other donors, and what prompts them to do it? I’ve got some donors who have donated, and I don’t know why they are donating – they are donating to fill a need, to fill a gap, to feel grief, to feel valued, or something?*”

*“If you’ve got a mother lactating for some period of time, do you…insist that she’s “engaged” with someone for some support, whether it’s a GP, bereavement counsellor…is that part of the screening process, almost – seeing somebody?”*

*“The women are doing it because they can, it’s the same, it probably gives them some value that they can produce this milk…it’s altruistic.”*

Women’s autonomy with regard to milk donation was recognised by all Stakeholders as complex and as a topic it occupied the bulk of the discussion. Autonomy is a respect for personal self-government and recognition of the role an individual plays in making decisions regarding one’s own body [16,17]. Decision-making and autonomy is of particular relevance to each bereaved mother’s lactation decision after neonatal death. As a consequence of becoming a donor, Stakeholders recognised that lactation decisions also needed to be considered in relation to HMB’s donation guidelines. Milk donation to HMBs in Australia is voluntary and unpaid [3]. Once accepted as a donor, women may choose the length of time they may donate within the specific limits set by the HMB. Decisions about lactation and donation are therefore not only informed by the donor herself, but may be negotiated in conjunction with interactions with HMB staff and the donation guidelines.

Stakeholders’ discussed the shape bereaved milk donation programs may take in terms of inclusion criteria, and how these programs may differ (or not) from milk donation for mothers with living infants. In particular, Stakeholders deliberated on the length of time after infant death that women could donate milk to a HMB, and who should determine this. Currently some milk banks in Australia place a six-month limit on the donation period from all donors. That is, donors can provide milk to the HMB until their baby reaches the age of six months, while others have a more flexible donation period. Stakeholders debated whether there should be a specified length of time that may or may not differ from that allowed to milk donors with living infants. For example, one milk bank representative talked about their HMB’s current policy for bereaved donation that supports mothers to work toward involution after neonatal death. In this particular HMB women are encouraged to provide their milk in the days to weeks following the death of their baby. However this particular HMB had a policy that distinguished between milk donated as a result of milk suppression (which they deemed as acceptable), and the milk actively produced through deliberate expression to sustain lactation where there is no surviving infant, which was not supported. This particular milk bank was concerned about the possibility that sustaining lactation in the absence of a surviving infant may negatively impact the grieving process for a mother, although there is no research to support or refute this position. The staff at this particular HMB also held concerns that milk banks currently may not have the resources to identify, assess and manage bereavement issues appropriately. Some issues that milk banks may need to consider with respect to these donations are; changes in milk composition that have been shown to occur during weaning and where supply drops below 300 ml/day [18] and impacts on family planning and relationships e.g. lactational amenorrhea [19]. To date, research has not examined these issues and as such this HMB decided that it was impossible for them to assess the suitability this type of donation and the suitability of the donated milk for the intended recipient.

As a counterpoint, many Stakeholders questioned whether it was equitable if one mother with a living infant could donate for 6 months, while a bereaved mother is told that she cannot. One Stakeholder stressed the need to recognise the equality and parallels between bereaved mothers donating milk and non-bereaved mothers donating milk:

*“And I think to say, ‘grief ends at three months. I don’t want any more from you at three months!’ – I can’t say that! Especially when I’m accepting donations from other women up to a year”*.

Stakeholders considered this ethical difficulty and whether different guidelines for the bereaved versus non-bereaved donor would manifest as discriminatory, paternalistic or judgemental in terms of what is ‘normal’ lactation. While acknowledging the particularly complex emotional and psychological experiences of bereaved mothers, all Stakeholders acknowledged that a woman’s mental, emotional and physical health was of upmost importance, but that this was a standard held for all donors, and not just those who may be bereaved.

Stakeholders suggested that milk donations from bereaved mothers could be accepted by the HMB for as long as a woman feels comfortable, so long as that fits within the guidelines of the HMB. Moreover they suggested that the milk donated by bereaved mothers could be as a result of suppression, from frozen milk stores held in the NICU or at home, or as a result of bereaved mothers’ decisions to continue expressing after infant death. One Stakeholder felt very strongly that more research is required on the psychology and emotion of sustaining lactation after infant death, including any potential risks to the donor, before they could support donation of milk expressed after infant death. Broadly, there was support among Stakeholders to encourage women to eventually move toward not expressing milk but that this end point in terms of timing should be flexible for each woman.

(iv) ** Discussing donation with bereaved mothers**

“*We’ve never approached mothers about donating their milk after the loss of a baby; they’ve actually approached us*.”

“*People would find it a very awkward conversation to have*”

“*I think a lot of nurses and midwives would probably find the subject of continuing demand in milk quite a challenging one*.”

“*They might have been a midwife and not game to bring up the subject. And that’s something we could do a lot better*.”

Many Stakeholders used stories to illustrate the difficult nature of bringing up the topic of lactation and donation in conversation with the parents after neonatal death. It was recognised as “very awkward”, “quite challenging”, and one that many were “not game” to bring up. However, Stakeholders also recognised the necessity of doing so, and acknowledged that this was “something we could do a lot better”. Stakeholders agreed that an appropriate time to discuss the topic of donation could be during the milk suppression conversation, thus offering a valid alternative to the bereaved mother.

The Stakeholders identified that the ideal person to discuss lactation management and donation with bereaved mothers would be the primary carer of the mother and infant, or someone who has built rapport with the mother within the NICU as the first point of contact after neonatal death. Stakeholders stipulated that contact with the mother in the form of support (psychological, physical and specifically about lactation) needed to occur within the first four hours of the infant’s death. Stakeholders also acknowledged the importance of support from a lactation team, demonstrating that a collaborative approach was ideal. Stakeholders were also unanimous in their agreement that the baby’s death needed to be recognised in this conversation, and that currently this was not adequately addressed.

(v) ** Spread too thin: A lack of Human Milk Banks in Australia**

*“We decided that we couldn’t support [extended milk donation after neonatal death], we didn’t feel like our milk bank had the skills to ensure that it was the best thing for the mum.”*

*“We didn’t feel like we were in a position to manage that situation appropriately. We certainly don’t try to push long term donations as ‘abnormal’, we just try really to support the parents in finding what ‘fits’”*

*“I’m just worried we are going to open a floodgate that we aren’t going to be able to control.”*

Stakeholders all stated that the donor’s welfare was of primary concern, and that significant resources were required to invest in donor screening, and milk handling, pasteurisation and storage. Stakeholders articulated that HMBs are often already ‘spread too thin’ and that additional resources and networks were required in order to ensure financial viability with regard to screening bereaved mothers and their (typically) lower volumes of milk, in addition ensuring that correct psychological supports were in place for bereaved donors, should it be needed. Many Stakeholders recognised that there was tension between ensuring a duty of care to all donors and the costs associated with this in terms of the HMB’s core operating functions. One Stakeholder stated that they did not feel the HMB in isolation had the skills to ensure the best outcome for bereaved mothers and that they were not in a situation to manage bereaved mothers appropriately. Other Stakeholders reiterated that the support provided to bereaved donors was the same individualised care provided to each donor, and that this was what was already happening in practice. Stakeholders recognised the need for resources such as bereavement counsellors or GPs to be part of the network that HMB staff utilise to ensure adequate support.

There are relatively few HMBs in Australia and some face tight governmental restrictions that prevent accepting frozen stores of breast milk such as that which may be donated by a bereaved mother. Therefore Stakeholders raised the importance of drafting national guidelines to advise bereaved mothers on lactation management in cases where there is no HMB available to them to donate, or when they do not meet eligibility criteria. Stakeholders agreed that in most of these circumstances women would suppress their lactation with advice and support from lactation support health professionals and breastfeeding counsellors in the community. The majority of Stakeholders agreed that a woman may be supported to express if she wanted to experience lactation, and that this milk could be saved as a memento of her infant. It is also important to acknowledge that if there were a milk bank, that this milk could have been donated. HMB staff are aware of the increasing popularity of online milk sharing [20-22], and recognise that bereaved mothers who do not meet HMB criteria may wish to donate their milk in this way. However, Stakeholders expressed their concern regarding the safety concerns associated with private/online milk sharing.

## Conclusion

Twelve key Stakeholders involved in milk banking and milk donation attended Australia’s first NSM on the topic of milk donation after neonatal loss. During the NSM Stakeholders heard the latest research on milk donation after neonatal death, reviewed bioethical principles and had the opportunity to identify, discuss and improve upon existing HMB and NICU donation practices that may be specific to the needs of bereaved mothers. Several practice issues were identified during the NSM, and key considerations for bereaved milk donation programs in Australian HMBs were discussed (Table 3). While these considerations were drawn from empirical data specific to the NICU and bereavement, we believe that these results are applicable to donation by bereaved mothers with older infants.

**Table 3:** **Stakeholder identified key practice issues for milk donation after neonatal death**

**Theme**	**Implications for practice**
The spectrum of milk donation	Accept that all women experience lactation and donation differently
The quality of donated milk	Milk banks are responsible for providing safe and appropriate breast milk to recipients
Caring for the bereaved donor	Accept that all women grieve differently
	Ensure that bereaved mother’s welfare is a priority
	Suggest broader support networks for bereaved mothers who are donors
Women’s autonomy	Attend to the commonalities between bereaved mothers and non-bereaved mothers as donors when screening and assessing suitability, while accommodating the special needs associated with bereaved mothers
	Avoid judgement of women’s lactation and donation decisions after infant death
Approaching bereaved mothers about donation	Provide bereaved mother with the option of milk donation, when available
Spread too thin	Provide donation guidelines or information on further support in cases where no HMB is available or if donation does not fit within guidelines
Four categories of milk donation after neonatal death	Bereaved mothers have different patterns of milk donation. These may need to be considered with regard to individual milk banking guidelines. (i) donation of previously expressed milk/frozen stores (ii) donation as a result of sustained lactation where there is a surviving infant who is being fed by the expressed breast milk or through breastfeeding (iii) donation of breast milk that is expressed as part of lactation suppression, and (iv) donation of milk expressed during sustained lactation where there is no surviving infant

During the course of the NSM it became clear that there are four distinct categories of donation after neonatal death which influenced the discussion of practice and the group’s ability to reach consensus: (i) donation of previously expressed milk/frozen stores (ii) donation as a result of sustained lactation where there is a surviving infant who is being fed by the expressed breast milk or through breastfeeding (iii) donation of breast milk that is expressed as part of lactation suppression and, (iv) donation of milk expressed during sustained lactation where there is no surviving infant. There was general national Stakeholder agreement that there may be a role for HMBs to accept milk from the first three categories of milk donation. However, although Stakeholders recognised that some donors generously offer the fourth category of milk, agreement among Australia’s Stakeholders was not achieved regarding the appropriateness of this donation due to one Stakeholder’s concern for the well-being of the donor, her family, and the composition of the milk that would be donated. Further research is required to determine the psychological impact and social well-being experienced by mothers and their families as a result of all forms of milk donation, and further exploration is required in order to offer optimal milk donation programs to all bereaved mothers.
